# Sex-specific metabolic profiling to explain the increased CVD risk in women with migraine: a narrative review

**DOI:** 10.1186/s10194-023-01601-5

**Published:** 2023-06-06

**Authors:** F. C. van Welie, L. A. Kreft, J. M. A. Huisman, G. M. Terwindt

**Affiliations:** Department of Neurology, Leiden University Medical Center, P.O. 9600, 2300 WB Leiden, The Netherlands

**Keywords:** Cardiovascular disease, Cerebrovascular disease, Metabolomics, Biomarkers, Lipids

## Abstract

**Background:**

Migraine is a disabling neurological disorder whose diagnosis is based on clinical criteria. A shortcoming of these criteria is that they do not fully capture the underlying neurobiological factors and sex-specific complications in migraine such as cardio- and cerebrovascular disease. Biomarker research can help to improve disease characterization and identify pathophysiological mechanism underlying these comorbidities.

**Objective:**

In this narrative review we searched for sex-specific metabolomics research to identify markers that may explain the migraine-cardiovascular disease (CVD) relationship.

**Discussion:**

Large-scale plasma metabolome analyses revealed alterations in migraine. Sex-specific findings showed a less CVD-protective HDL metabolism as well as the ApoA1 lipoprotein, especially for women with migraine. To explore other possible pathophysiological pathways, we expanded our review to include inflammatory markers, endothelial and vascular markers and sex hormones. Biological sex differences may affect the pathophysiology of migraine and its complications.

**Conclusions:**

There is no general large dyslipidemia profile in migraine patients, in line with findings that the increased risk of CVD in migraine patients seems not to be due to (large artery) atherosclerosis. Sex-specific associations are indicative towards a less CVD-protective lipoprotein profile in women with migraine. Future studies into the pathophysiology of CVD and migraine need to take sex specific factors into account. By establishing the overlapping pathophysiological mechanism of migraine and CVD, and unraveling the associated effects these diseases exert on each other, better preventative measures can be identified.

## Key points

• There is no general large dyslipidemia profile in migraine patients, in line with findings that the increased risk of CVD in migraine patients seems not to be due to (large artery) atherosclerosis.

• Sex-specific associations are indicative towards a less CVD-protective lipoprotein profile in women with migraine.

• Future studies into the pathophysiology of CVD and migraine need to take sex specific factors into account.

## Introduction

Migraine, a disabling neurological disorder, affects over 1 billion people worldwide, with women experiencing two to three times higher prevalence than men [1]. In addition to this, migraine has been shown to be associated with a higher risk of cardiovascular and cerebrovascular events [2]. Despite its high impact on people’s life and society this debilitating disorder does not seem to receive as much attention when compared to other neurological diseases [3]. Understanding the mechanisms behind migraine attacks, with paying attention to sex-specificity, will help to understand sex differences in migraine and its related cardio- and cerebro-vascular disease (CVD) comorbidities. These efforts may help to develop safer and more effective treatments for migraine patients.

### Migraine pathophysiology and the role of sex hormones

**Fig. 1:**
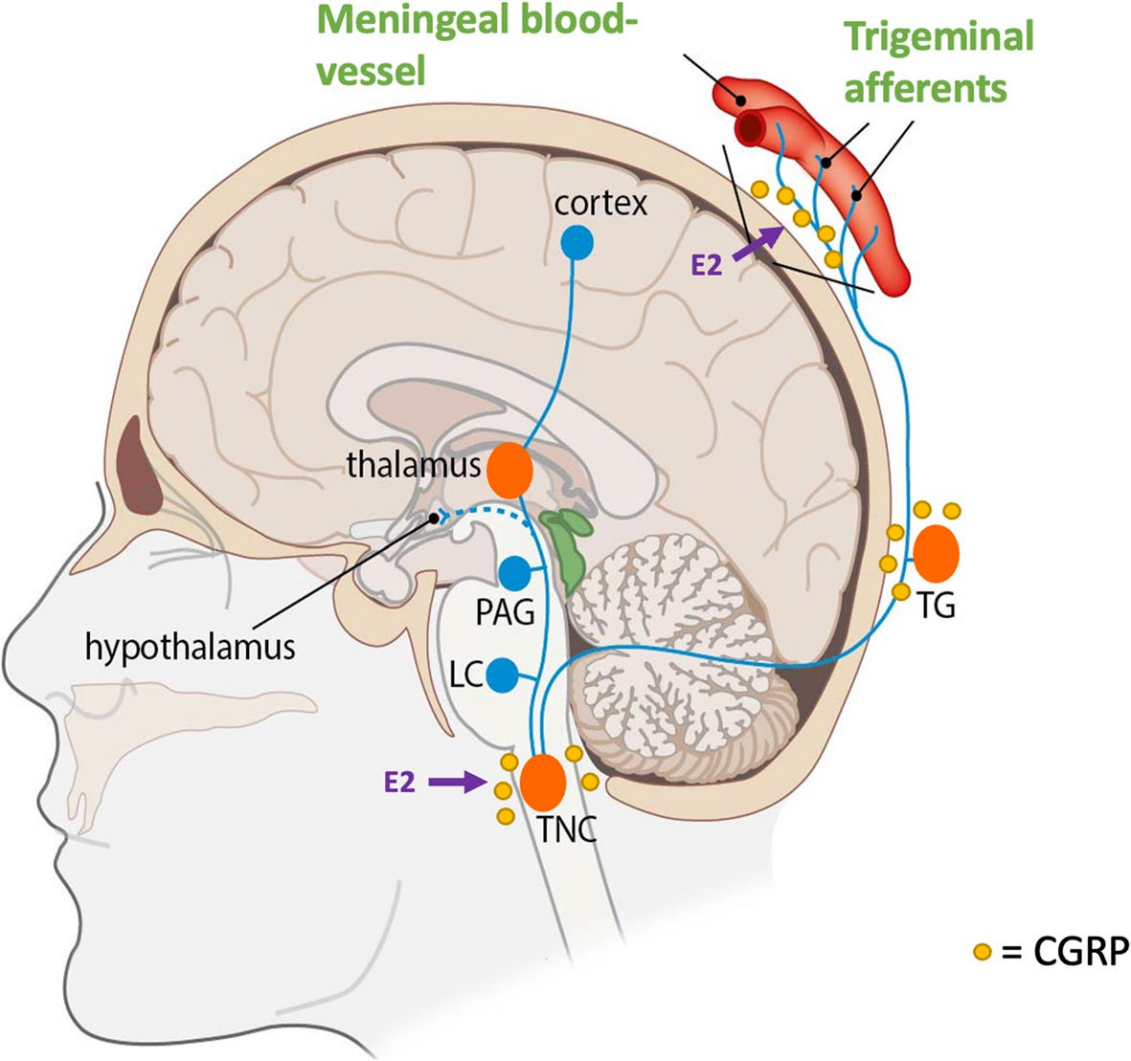
**The activated trigeminovascular system during a migraine attack.** When the trigeminovascular system is activated during the headache phase of a migraine attack, the trigeminal afferents, the trigeminal ganglion (TG) and the trigeminal nucleus caudalis (TNC) in the brainstem are activated. The involved neurotransmitter is calcitonin gene related peptide (CGRP). The sex hormone estrodial (E2) influences the CGRP release in the TNC and the trigeminal afferents in humans [8]. Notably, in rodent studies it has been shown that E2 also effects the CGRP release in the TG, which is not specified in this image as this image shows a human model [8]. Copyright: Leiden Headache Center, LUMC, NL

The headache phase during a migraine attack is caused by activation of the trigeminovascular system, see Fig. 1 [4]. The trigeminal nerve has nociceptive trigeminal afferents surrounding cranial blood vessels. It is assumed that the trigeminovascular system can be activated by cortical spreading depolarization (CSD). This is a depolarization wave over the cortex, in which neurons fire briefly and then are exhausted and is seen as the electrophysiological substrate for the migraine aura. However, an aura can also occur without headaches and many migraine patients never experience an aura. Thus, CSD may not necessarily be the cause of activation of the trigeminovascular system. When the trigeminal nerve is triggered, the trigeminal afferents, the trigeminal ganglion and the trigeminal nucleus caudalis in the brainstem are activated. During this signal transmission calcitonin gene related peptide (CGRP), a powerful vasodilator, acts as an important neurotransmitter [5, 6]. Interestingly, CGRP can induce migraine-like attacks in humans [7].

Clinical and epidemiological studies suggest a prominent role for sex hormones in female migraine patients. Menstruation is an important factor that lowers the threshold for getting a migraine attack [9]. Hormonal fluctuations during perimenopause are also accompanied by increased susceptibility to migraine attacks [10]. In contrast, an improvement in migraine without aura (MO) is often reported during pregnancy [11]. Thus, sex hormones are known to affect susceptibility to migraine attacks in women, but there is still insufficient understanding of the underlying pathophysiological mechanism. It is believed that the rapid drop in estrogen prior to menstruation may be the trigger for migraine attacks [12].

It has been suggested that effects of CGRP may be affected by sex hormones. The hypothesis might be that high stable levels of estrogen cause a suppression of the CGRP system and stimulation of the oxytocin system, although this latter has not been tested yet [13–16]. Thus, the chance of a migraine attack may then be lower. During menstruation or the stopping week of the contraceptive pill, plasma levels of estrogen and oxytocin decrease, which may trigger an activation of the CGRP system, what may cause activation of the trigeminal nerve and increase the likelihood of a migraine attack [16]. Experimental animal data suggest that estrogen modulates differently, and in a complex manner, the density of CGRP receptors in the peripheral and central nervous systems. Further studies are clearly required to determine the exact role of estrogens, and which estrogen receptors are involved, in the modulation of CGRP expression in the different components of the trigeminovascular system [8]. Furthermore, androgen and progesterone receptors are also expressed in the different components of the trigeminovascular system. Since studies with these hormones are very limited, the role of androgen and progesterone receptors in modulating CGRP in the trigeminovascular system remains to be determined [8].

### Cardio- and cerebrovascular risk of migraine

As stated earlier, migraine is associated with a higher risk of cardiovascular and cerebrovascular events, which was shown by a large meta-analysis study [2]. This was mainly caused by a higher risk for a stroke or heart attack. Migraine is an independent risk factor for having a cerebral and/or myocardial infarction [17, 18]. The risk of cerebral infarction is 2.5 times higher in young women (< 55 years of age) with MA compared to women without migraine. This relative risk increases to a 35 times increased risk if a young female with migraine also smokes and uses the contraceptive pill, although the absolute risk remains low for young females [19–25]. In addition, in a Dutch population-based MRI study, migraine patients had an increased risk for white matter lesions and cerebellar infarcts, most pronounced in women with MA [26]. In the follow-up study after 9 years, it was found that migraine patients had more progression of white matter lesions compared with controls, independent of migraine activity [26, 27]. There was no significant association of high vs nonhigh deep white matter hyperintensity load with change in cognitive scores (-3.7 in the migraine group vs 1.4 in the control group; 95% CI, -4.4 to 0.2; adjusted *P*= 0.07) [27]. White matter hyperintensity are common in migraine especially in those who are older and have aura, and this MRI study has been replicated in other cohorts [28–30].

It is hypothesized that the migraine-CVD association may be due to joint pathophysiological mechanisms, in which the migraine and CVD are manifestations of vascular dysfunction. CVD etiology in women seems to involve a wider spectrum than atherosclerotic large vessel disease which is the most common etiology in men [31]. In ischemic heart disease and stroke, an increased focus on female type pathophysiology revealed a large role for microvascular, endothelial and vasomotor dysfunction as opposed to the obstructive atherogenic pathophysiology more often observed in men. Thus, it has been suggested that for women the migraine-CVD association might not be explained by the more traditional vascular risk factors that would lead to atherosclerosis [32, 33]. Genetic factors probably also play a role. In genome-wide association studies vascular risk factors were shown for migraine which overlap with vascular disorders and, in addition, the role of CGRP was also highlighted in these studies [34, 35]. After an ischemic event, CGRP is known to play a protective role in the regulation of vascular tone [32, 33]. CGRP may also contribute in preventing cardiac ischemia in women, because higher CGRP levels were observed when compared to men [36, 37].

Despite the recent advances there is still much unknown about the link between CVD and migraine and, particularly, how this relates to sex-differences. Biomarker research, particularly metabolomics studies, may help to improve disease characterization and identify pathophysiological mechanism underlying these comorbidities. The sex specificity of migraine and the associated increased CVD risk is likely indicating pathophysiological dissimilarities between men and women. Besides the existence of factors that are unique to women such as sex hormones we hypothesize that other risk factors, such as lipid or other metabolomic profiles may also be different in women with migraine. In this narrative review, we will discuss sex-specific conducted metabolomics research, with a focus on lipoprotein platforms, to identify markers that may explain the migraine-CVD relationship. In addition, we studied other possible pathophysiological pathways by looking at different types of markers, namely: inflammatory markers, endothelial and vascular markers, and sex hormones. Finally, we provide with a discussion on the current findings and recommendations for the future.

## Lipoprotein profile

As mentioned in the introduction, we hypothesize that lipids or other metabolomic profiles may be different in individuals with migraine compared with healthy controls but also for women with migraine compared to men with migraine. These differences could be the underlying cause for the increased risk for CVD in women with migraine especially. Therefore, we focused on studies conducted on lipid metabolite markers in migraine patients. These studies are summarized in Table 1.

**Table 1:** Overview of the mentioned lipoprotein papers presenting the set-up, techniques, cohort and main results

Authors	Type of study	Technique	Participants	Main findings
Onderwater et al. [38]	Cohort	^1^H-NMR spectroscopy	Total: 10,153, males and females analyzed separately • 2800 migraine patients • 7353 controls	• ↓ ApoA1 in both males and females • ↓ S-HDL-FC ratio in both males and females • ↓ Omega-3 fatty acids in males
Harder et al. [39]	Cohort	^1^H-NMR spectroscopy	Total: 1825, males and females analyzed concurrently • 313 migraine patients • 512 controls	• Several metabolites found either as predictive for lifetime migraine status (6) or for active migraine status (22)
Liampas et al. [40]	Meta-analysis	Enzymatic assays or colorimetric method, standard laboratory methods	12–15 studies with a total ranging from 2538–2889, males and females analyzed concurrently • 1325–1526 migraine patients • 1213–1363 controls	• ↑ TC and TG levels in migraine patients versus controls • Similar HDL-C levels in migraine patients versus controls
Kurth et al. [41]	Cross-sectional	Immuno- turbidimetric assays and reagent assays	Total: 27,626 exclusively females • 5087 migraine patients • 22,539 controls	• No significantly decreased apoA1 levels in women
Bellei et al. [42]	Cross-sectional	Two-dimensional gel electrophoresis and mass spectrometry	Total: 45 exclusively females • 30 migraine patients • 15 controls	• ↓ ApoA1 was found in female migraineurs (menstrual and post-menopausal migraine)
Maghsoumi-Norouzabad et al. [43]	Meta-analysis	Not specified	For duration of migraine: 2 studies with a total of 134 males and females analyzed concurrently • 62 migraine patients • 72 controls	• Duration of migraine decreased with use of omega-3 supplements

Large population-based studies showed elevations of total cholesterol, low-density lipoprotein cholesterol (LDL-C), and triglyceride levels, and decreased high-density lipoprotein cholesterol (HDL-C) levels, to be associated with migraine [44]. However, results were not consistently replicated due to methodologic variability such as cohort variability, measurement of crude lipoprotein levels, or studies failed to detect significant differences due to lack of statistical power. Two studies adopted ^1^H-NMR metabolomics, whereas other studies utilized more standard laboratory assays. Also, most studies did not specifically examine sex-differences in lipoprotein profiles. This emphasizes the need for a more systematic approach with a large lipoprotein platform and large cohort that have sufficient power, to ensure that the findings are valid, and taking sex differences into account [44].

Proton nuclear magnetic resonance (^1^H-NMR) allows for the rapid simultaneous identification and quantification of hundreds of metabolites measured in body fluids. ^1^H-NMR can provide metabolic profiles in large patient cohorts with more detailed pathophysiologic insight, compared to the traditional and limited numbers of blood-based lipid measurements such as cholesterol, LDL, HDL, and triglyceride levels [45].

**Fig. 2:**
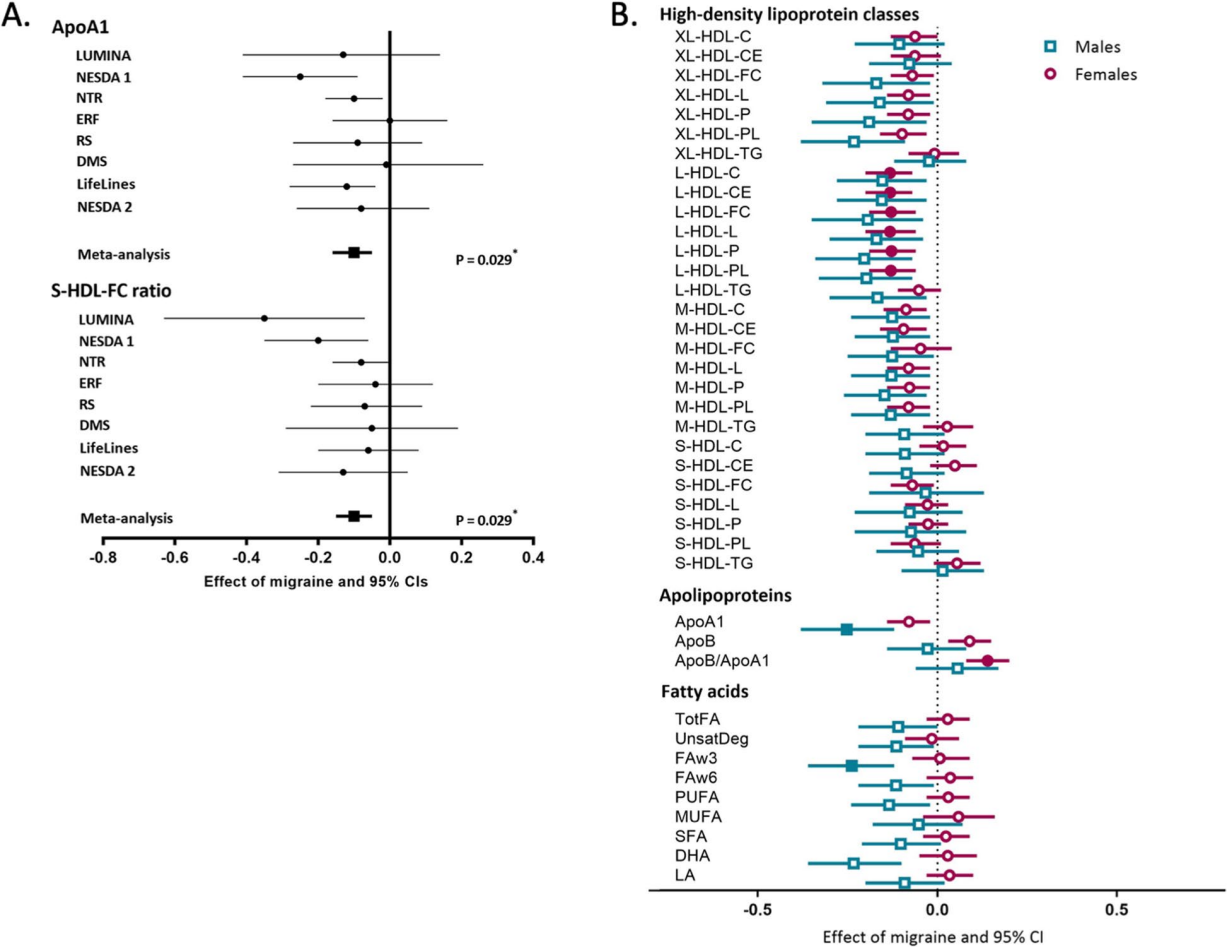
**Results of the meta-analysis performed by Onderwater et al.** [38] **A.** **Candidate migraine biomarkers apolipoprotein A1 (apoA1) and the free cholesterol to total lipid ratio in small high-density lipoprotein ratio (S-HDL-FC).** Forest plot of the random-effects meta-analyses of the associations with migraine. The effect sizes and 95% confidence intervals (CIs) for apoA1 and S-HDL-FC are shown per cohort. Values from logistic regression with migraine status as dependent variable and metabolite levels, age and sex as independent variables. *p Values after Holm-Bonferroni multiple testing correction (*p* < 0.0002). I^2^ = measure of heterogeneity; LUMINA = Leiden University Migraine Neuro-Analysis; NESDA = Netherlands Study of Depression and Anxiety 1&2; NTR = Netherlands Twin Registry; ERF = Erasmus Rucphen Family study; RS = Rotterdam Study. **B.** **Sex-stratified metabolite associations with migraine.** Random-effects meta-analysis of 8 cohorts of the metabolite associations with migraine in male (blue squares) and female (purple circles) participants. The effect sizes and 95% confidence intervals (CIs) are presented. The solid squares or circles indicate significance after Holm-Bonferroni multiple testing correction (*p* < 0.0002)

In a large systematic study plasma samples from eight Dutch cohorts, a total of *n* = 10,153 subjects (2,800 migraine participants and 7,353 controls), were profiled on a 1H-NMR-based metabolomics platform, to quantify 146 individual metabolites (e.g., lipids, fatty acids, and lipoproteins) and 79 metabolite ratios [38]. This study identified two circulating candidate migraine biomarkers, which are both related to HDL status: a decreased level of apoA1 (an apolipoprotein with a specific association with HDL) and a decreased S-HDL-FC ratio (the free cholesterol to total lipid ratio in small HDL). In addition, only in male participants fatty acids of the omega-3 class were shown to be associated with migraine. Many other positive and negative correlations could be observed and possibly also sex differences, however only the three metabolites mentioned above were deemed significant (see Figs. 2a and b). Global test analysis further supported that HDL traits (but no other lipoproteins) were associated with migraine status. This study also assessed the influence of smoking, fasting status, depression, lipid-lowering medication usage, and BMI on apoA1 levels and the S-HDL-FC ratio. Small effects of lipid-lowering medication usage and BMI on apoA1 and S-HDL-FC ratio plasma levels were identified. Other covariates did not influence these levels. For all cohorts, BMI and lipid-lowering medication usage were subsequently added to our model. The expanded model revealed that a decreased apoA1 level and S-HDL-FC ratio were still associated with migraine. To further support the robustness of the candidate biomarkers, correlation analyses using samples from a small number of participants, acquired on two occasions revealed particularly stable results between measurements in the same individual patient for apoA1 and to a lesser extent for S-HDL-FC ratio.

Possible differences in the metabolite profile associated with migraine between male and female participants were analyzed. ApoA1 levels were significantly associated with migraine in male participants, with smaller effects, but in similar direction, in female participants. Furthermore, the apoB/apoA1 ratio was significantly higher in female migraineurs compared to female controls. The S-HDL-FC ratio was negatively associated with migraine in female participants but failed to reach significance after correction for multiple testing. In male participants, no apparent relation was identified for the S-HDL-FC ratio. Associations with lower medium and large HDL measures were significant in female participants, with a similar finding in male participants, although not significant. Interestingly, in male participants, lower omega-3 fatty acids were associated with migraine, an association not seen in female participants.

In summary, this large ^1^H-NMR-based metabolomics study showed a sex-specific decrease in apoA1, with especially lower values in males with migraine but not in women [38]. ApoA1 is the main apolipoprotein of plasma HDLs and is shown to be cardioprotective [46]. Interestingly, in male participants, lower omega-3 fatty acids were also associated with migraine, an association not seen in female participants. Female migraine participants showed lower medium and large HDL measures compared to female controls. These sex-specific findings may lean towards a less CVD-protective lipoprotein profile in female migraine participants compared to males with migraine [38].

Of note, omega-3 fatty acid supplements, due to their anti-inflammatory action, have been investigated in migraine attack prevention. A meta-analysis suggested a reduction on the duration of migraine attacks, when using omega-3 fatty acid supplements [43]. But no significant effect was found for the frequency or severity of the attacks. Also, the number of participants in these studies were small, the analysis for the duration of migraine had a total of 134 participants.

Notably, in line with the above findings, the large Women’s Health Study observed no significant decreased apoA1 levels in 5,087 female participants with a history of migraine (total population 27,626) [41]. However, compared with women with no migraine history, women who reported any history of migraine had modestly elevated total cholesterol, non-HDL-C, ApoB100. The authors suggested that this increase did not meaningfully differ according to MA status and migraine frequency, although it may be questioned whether this study was designed for sub-analyses on migraine subtype and frequency, as aura symptoms and frequency may vary and are often not reported in detail.

Another very small study analyzed in menstrual-related migraine (*n* = 15), menopausal migraine (*n* = 15) and non-headache (*n*= 15) women the serum profile, wherein apoA1 was found to be decreased in the migraine women, but no robust data could be presented because of the small sample size [42]. Contrasting results were found in a meta-analysis, in which the authors determined that TC and TG levels were found to be higher in migraine patients (both sexes were analyzed concurrently), whereas very similar HDL-C levels were found in migraineurs and controls [40]. Initially, they also found elevated LDL-C levels in migraine patients, but after exclusion of studies not presenting comparable BMI levels between patients and healthy controls, this was established as non-significant.

From a different perspective is the study on metabolite profiles using ^1^H-NMR in serum samples of migraine patients and controls from the Erasmus Rucphen population [39]. The aim was to identify and quantify metabolites in serum that are associated with lifetime and active migraine (migraine attacks in the last year) by comparing metabolic profiles of patients and controls, regardless of their sex, although a majority of participants were female. Fasting serum samples from 313 migraine patients and 1512 controls from the Erasmus Rucphen Family (ERF) study were available for 1H-NMR spectroscopy. In this study, profiles consisting of six metabolites were predictive for lifetime migraine status and profiles of 22 metabolites were predictive for active migraine status. Several of the metabolites in this profile were involved in lipid, glucose and amino acid metabolism. This study indicates that metabolic profiles, based on serum concentrations of several metabolites, including lipids, amino acids and metabolites of glucose metabolism, can distinguish active migraine patients from controls. The cholesterol and lipids that were found to be different in migraine patients compared to controls suggested some dyslipidemia in migraine patients [39].

In conclusion, there is no general large dyslipidemia profile in migraine patients, in line with findings that the increased risk of CVD in migraine patients seems not to be due to (large artery) atherosclerosis. Importantly, however, there are sex-specific associations that indicate a less CVD-protective lipoprotein profile in female migraine participants.

## Other markers

### Inflammatory marker CRP

There is evidence that inflammation can be caused by endothelial damage in migraine. This may be implicated in endothelial dysfunction as an explanation for migraine and the increased risk for stroke [47]. Therefore, in this section we will discuss inflammatory and endothelial or other vascular markers in migraine. An important inflammation marker that was suggested to be linked to migraine is C-reactive protein (CRP) [48]. CRP is an acute phase protein and a highly sensitive non-specific marker for inflammation, as its circulating levels rise during an inflammatory response. However, it is important to note that CRP is not specific to any one condition or disease and cannot definitively diagnose a specific disease. Elevated CRP levels can indicate a range of inflammatory conditions, but can also be present in otherwise healthy individuals who exercise regularly or have certain genetic variations. Relying too heavily on CRP as a diagnostic tool can be problematic due to factors that affect CRP levels, such as age, gender, smoking status, and medication use. Significant variability in CRP levels among individuals means that a single measurement may not accurately reflect baseline levels or changes in response to treatment. Therefore, interpreting CRP levels should always consider a patient's medical history, physical examination, and other diagnostic tests.

Information on cross-sectional interictal CGP levels in migraine are inconsistent but some studies have reported a cross-sectional association between elevated high sensitivity C-reactive protein (hs-CRP) and migraine. Interestingly, a large health study investigated the influence of high-sensitive CRP (hs-CRP) at baseline on the risk of developing (chronic) migraine 11 year later. A total of 19,574 participants without migraine at baseline were divided into three groups based on hs-CRP levels (< 3 mg/L, 3–9.99 mg/L and 10.00–20 mg/L). Increased risk of migraine (RR 1.46, 95% CI 1.05–2.04) was found in the highest hs-CRP levels group compared to the lowest group. In the group with the highest hs-CRP levels, a nearly three times higher risk of chronic migraine (RR 2.81, 95% CI 1.12–7.06) was found, whereas no evident relationship was found between high hs-CRP level and risk of developing episodic migraine [49]. What makes CRP levels in migraine interesting is the fact that CRP has also been established as a predictor for CVD [50], and there seems to be a sex difference in the relation of CRP with CVD. It would be interesting for future research to investigate if the association of CRP with migraine also differs between men and women.

### Endothelial and vascular markers

Migraine is not only associated with cardiovascular disease but also with stroke. It has been observed that being a women with high attack migraine frequency and a long migraine duration increases the stroke risk [17, 51]. Endothelial dysfunction has been suggested as a possible underlying shared mechanism for this increased risk for cerebrovascular diseases [44]. In a large population-based case–control study, endothelial and vascular biomarkers were compared between migraine patients and controls [44]. Fibrinogen was elevated in the total migraine group and the subgroup migraine with aura versus controls and hs-CRP for the total migraine group. When logistic regression analysis was applied, elevated levels of fibrinogen, factor II, band hs-CRP were more likely to occur in those with migraine and the migraine aura subgroup. Elevated fibrinogen was also more likely in migraine without aura. After correction for factors predictive of the examined biomarkers, the results for fibrinogen and factor II remained significant. Interestingly, In a small cross-sectional trial the fibrinogen quotient (cerebrospinal fluid/plasma fibrinogen ratio) in migraine patients (*n* = 42) was higher than in controls (*n*= 14) [52]. In a recent publication, a mendelian randomization analysis was performed to assess a relationship between several hemostatic measures and migraine susceptibility [50]. Twelve blood-based measures of hemostasis were examined, including plasma level or activity of hemostatic factors and fibrinopeptides [50]. The findings supports potential causality of increased FVIII, vWF, and phosphorylated fibrinopeptide A, but surprisingly for decreased instead of increased fibrinogen in migraine (especially with aura) susceptibility [50].

Endothelin-1 (ET-1) is a peptide comprising 21 amino acid residues derived from the endothelium. Previous studies have proposed a possible link between ET-1 and migraine. Specifically, increased ET-1 levels have been noted during the ictal phase of migraine. Moreover, they induce cortical spreading depression in rats [53]. In a small study interictal plasma endothelin-1 levels were not different among participants with episodic migraine (*n* = 87), with chronic migraine (*n* = 88), and controls (*n* = 50). Endothelin-1 levels did not differ among participants with migraine with aura, those with migraine without aura, and controls (9.36 [4.26–13.35] vs. 9.50 [7.23–13.02] vs. 9.46 [7.00–14.19], *p* = 0.975). Thus, Interictal plasma endothelin-1 level is an unlikely marker for migraine.

Endothelial microparticles (EMPs) are vesicles that are released from activated endothelial cells and serve as a surrogate for endothelial dysfunction (ED). Stromal cell-derived factor-1 alpha (SDF-1α) is involved in the maintenance of endothelial integrity via mobilization of vascular stem cells. In a small study with women with MA and female controls EMP levels were elevated in women with MA associated with increased arterial stiffness assessed using fingertip tonometry and the heart rate-adjusted augmentation index (AI) [54]. Furthermore, the study observed reduced levels of SDF-1α in women with MA, which were associated with EMPs as a surrogate marker of endothelial dysfunction [55].

### Sex hormones

Estrogens increase the susceptibility to CSD, the underlying mechanism for aura in migraine, but also influence CGRP production in neuronal nuclei in the trigeminal ganglia. The hypothesis is that high stable levels of estrogen cause a suppression of the CGRP system and stimulation of the oxytocin system [16]. The drop of estrogen, for example prior to the menstruation or in peri-menopausal period is associated with a higher prevalence of migraine [9, 56, 57]. It seems that estrogen can modulate the activity of brain neurotransmitters and might be involved in the sensitization of the trigeminal neurons through mediators [58]. One study examined sex hormones in males with migraine. Despite the small sample size (migraine participants *n* = 18, controls *n*= 22), higher plasma estrogen levels in males with migraine with similar free testosterone levels were found, leading to a lower free testosterone to estrogen ratio in male migraine participants [59].

Mouse models for migraine often use males and females with ovariectomy [60]. Not many studies are conducted on the natural sex hormonal fluctuation in female mice. The menstrual cycle in mice is very short (4–5 days), and consists of four phases; proestrus, estrus, metestrus, and diestrus [61]. This causes many studies to use female mice with ovariectomy to avoid confounding effects [61]. In sex-hormone specific research female mice with ovariectomy are evaluated without and with additional estrogen treatment [62]. Familial hemiplegic migraine type 1 (FHM1), a severe migraine with aura variant, is caused by mutations in the CACNA1A gene. Mutant mice carrying the FHM1 R192Q mutation exhibit increased propensity for CSD. The CSD phenotype is stronger in female R192Q mutants and diminishes after ovariectomy. But it was also shown that orchiectomy reciprocally increases CSD susceptibility in R192Q mutant mice. Chronic testosterone replacement restores CSD susceptibility by an androgen receptor-dependent mechanism. Hence, both estrogens and androgens modulate genetically-enhanced migraine susceptibility [63]. Another study in mice with a natural estrous cycle that investigated the threshold for evoking CSD showed there was a lower threshold to evoke CSD during estrus stage which was characterized by significantly lower estradiol serum level [64].

## Discussion

There is no general large dyslipidemia profile in migraine patients, in line with findings that the increased risk of CVD in migraine patients seems not to be due to atherosclerosis. Sex-specific associations are indicative towards a less CVD-protective lipoprotein profile in women with migraine. Other possible pathophysiological pathways may include inflammatory markers, endothelial and vascular markers and sex hormones. It is important to realize that there are significant biological differences between men and women, which can affect the pathophysiology of migraine and its complications. Future studies into the pathophysiology of CVD and migraine need to take these sex specific factors into account.

There are several limitations to our narrative review. We performed a narrative review which takes a less formal approach than a systematic review in that it does includes the presentation of rigorous aspects characteristics of a systematic review such as reporting methodology, search terms, databases used, and inclusion and exclusion criteria. There are also several limitations in the current field of metabolomics for migraine. Firstly, most of the studies had small cohorts, which decreased the statistical power of findings. The results from different studies were contradictory to each other, however, different techniques were used. Because of a lack of studies employing metabolomics, we compared studies with varying techniques, but it should be noted that these studies are not directly comparable and require some nuance.

Recently, a few well-designed and well-powered studies on detailed lipid platforms have been performed. ^1^H-NMR is currently the more robust and reliable technique, thus studies using this technique have an advantage over other studies. In addition to the lipid profile, other possible mechanisms were also explored in this review. There is evidence linking inflammatory, endothelial and vascular biomarkers, and sex hormones to migraine. Sex differences have hardly been investigated yet for these platforms, which is surprising as migraine is more prevalent among women, indicating that sex differences at least have some influence on the occurrence of migraine. We strongly advise to take age, sex and pre/peri/menopausal status into account for metabolomic studies in migraine, especially for lipid platforms because after menopause the HDL-profile becomes less favorable for women [65].

Until recently, migraine could only be treated prophylactically with antihypertensive medicines, antidepressants, and antiepileptic drugs. These drugs may be effective but also have a list of potential side-effects. Recently, monoclonal antibodies (mAbs) against CGRP or its receptor have become available. CGRP and its receptors are present in the central- and peripheral nervous system and in the cardiovascular system (blood vessels and heart) [66]. Because of the wide expression of CGRP in the body, there have been concerns about the off-target effects of long-term inhibition. CGRP has a protective role in case of an ischemic event because of its role in vasodilation. There have been case reports suggesting a link between CGRP-mAbs use to migraine-related stroke and reversible cerebral vasoconstriction syndrome [67–70]. In addition, it has also been shown that patients who receive anti-CGRP medication are at risk to develop an increased blood pressure [71]. These findings highlight the importance of taking the risk for CVD in migraine patients into account when treating them.

Patients who suffer from migraine should be informed about the risk for CVD and a healthy lifestyle. Patients will ask questions on possibilities to reduce the CVD risk and whether lifestyle changes can help. The dietary supplements omega-3 polyunsaturated fatty acids (n-3 PUFAs) have been suggested to reduce migraine because of the anti-inflammatory activity. Human inflammatory cells contain high proportions of n-6 PUFAs and low proportions of n-3 PUFAs. However, if the intake of n-3 PUFAs is high enough they will replace the n-6 PUFAs in the cells. This shift interferes with processes in the cell involved in production of arachidonic acid-derived pro-inflammatory mediators such as prostaglandins. A recent small study suggested that a diet with higher pro-inflammatory properties, which is a diet associated with high serum inflammatory markers, was associated with an increased risk of migraine [72]. A pro-inflammatory diet has also been suggested to increase the risk for CVD [73, 74]. Also, for metabolic syndrome a pro-inflammatory diet had a stronger association in women compared to men [72].

### Conclusion and future trends

There is no general dyslipidemia profile in migraine patients. However, there are significant differences between males and females. Sex-specific associations are indicative towards a less CVD-protective lipoprotein profile in women with migraine. We also studied other possible pathophysiological pathways by looking at different types of markers, namely: inflammatory markers, endothelial and vascular markers, and sex hormones but hardly any sex specific metabolomic research has been performed. Therefore, we conclude that in future studies, sex differences must be considered when researching the relationship between migraine and CVD risk.

In the future, metabolomic studies, particularly those ^1^H-NMR-based, will play a major role in the discovery of relationship between migraine and CVD. For these studies, it is critical to include both men and women, but analyze their results separately. It is also important to look at different types of migraine, such as with and without aura, menstrual related migraine and take the fertile status of women into account. Discovering the pathophysiology through which migraine patients have an increased risk of CVD, could result in better preventative measures for these patients.

## Data Availability

Not applicable.

## Abbreviations

^1^H-NMR: Proton nuclear magnetic resonance
ApoA1: Apolipoprotein A1
BMI: Body mass index
CGRP: Calcitonin gene related peptide
CSD: Cortical spreading depolarization
CVD: Cardio- and cerebrovascular disease
HDL-C: High-density lipoprotein cholesterol
LDL-C: Low-density lipoprotein cholesterol
MO: Migraine without aura
MA: Migraine with aura
MM: Menstrual-related migraine
mAbs: Monoclonal antibodies
TC: Total Cholesterol
TG: Triglycerides
